# Pathogen Identification and Treatment of *Trichoderma koningiopsis* ZL01 Mycosis in Firefly *Pygoluciola* sp. (Coleoptera: Lampyridae)

**DOI:** 10.3390/insects16121193

**Published:** 2025-11-23

**Authors:** Yan-Hong Chen, Shi-Ling Wang, Fu-Xin Li, Lian-Bing Lin, Wei-Wei Li, Qi-Lin Zhang

**Affiliations:** 1 Faculty of Life Science and Technology, Kunming University of Science and Technology, Kunming 650500, China; 17777957759@163.com (Y.-H.C.); 15008728691@163.com (S.-L.W.); m18819776113@163.com (F.-X.L.); linlb@kust.edu.cn (L.-B.L.); 2 Yunnan Key Laboratory of Biodiversity Information, Kunming Institute of Zoology, The Chinese Academy of Sciences, Kunming 650201, China

**Keywords:** firefly larvae, mycosis, nystatin, *Trichoderma*, *Pygoluciola* sp.

## Abstract

The artificial breeding of fireflies is vital for supplementing natural populations. Unfortunately, mycosis is being observed with increasing frequency in the artificial breeding of fireflies, resulting in increased mortality. This study reports the identification of a microfungus that infects larval *Pygoluciola* sp., a species of semi-aquatic fireflies, during artificial breeding. Morphological and molecular analysis identified the fungi as *Trichoderma koningiopsis* (named as ZL01 strain). In addition, nystatin was selected out of five candidates as the optimal antifungal agent against *T. koningiopsis* ZL01, with a minimum inhibitory concentration of 25.00 μg/mL. Acute oral and contact in vivo toxicity tests on larval *Pygoluciola* sp. confirmed the safety of nystatin. Furthermore, compared to a *Pygoluciola* sp. larval population infected with *T. koningiopsis* ZL01, nystatin treatment increased the survival rate of larvae by twofold (spray administration) and threefold (drip administration) at the end of a nine-day artificial breeding experiment. These findings indicate that nystatin could be used as a potential antifungal agent to control mycosis in artificially bred fireflies. This study was the first to document the infection of semi-aquatic fireflies by pathogens and provide a corresponding treatment strategy.

## Simple Summary

Fireflies (Coleoptera: Lampyridae) have been artificially bred worldwide, and are frequently infected by microfungi during the long larval stage. In this study, we report a firefly pathogenic fungus, *Trichoderma koningiopsis* ZL01, isolated from larval *Pygoluciola* sp. Furthermore, nystatin solution was highly effective against *T. koningiopsis* ZL01 in vitro. Nystatin solution presented therapeutic efficacy and the absence of toxic effects for larval *Pygoluciola* sp. infected by *T. koningiopsis* ZL01. This study reports a rare method for treating fungal infection in fireflies and reducing the mortality of *Pygoluciola* species in artificial breeding.

## 1. Introduction

Fireflies are a common collective name for the beetle family Lampyridae (Insecta: Coleoptera), covering 100 genera and approximately 2200 species worldwide [1,2,3]. Fireflies are not only bioluminescent insects, but also possess considerable economic, cultural, and ecological value. Due to their high sensitivity to habitat degradation, they are considered important bioindicators used for the assessment of ecosystem health [4,5]. Natural firefly populations have experienced a dramatic decline recently due to habitat loss, degradation, light pollution, the overuse of pesticides, and other anthropogenic stressors. Raising fireflies indoors for release can be one of the conservation strategies [6].

Firefly adults are terrestrial, but their larvae can be classified into either terrestrial, semiaquatic, or aquatic lineages according to their habits [7,8]. *Pygoluciola* is recognized as a semi-aquatic firefly genus widely reared indoors, particularly in China [6]. Despite no reports of mycosis in the wild, fungal infections are frequently observed in the indoor rearing of firefly larvae, resulting in death before pupation. Thus, pathogenic fungi of fireflies is important to the indoor rearing. However, the diversity of fungi that are associated with fireflies is not well documented. The only study in the related literature reported the fungi that infect eggs (*Penicillium citrinum*) and larvae (*Trichoderma harzianum*) of *Pteroptyx bearni* during ex situ rearing project and the existence of *Trichoderma* found on fireflies [9,10]. Thus, identification of the pathogenic fungi and the development of treatment methods for mycosis are essential to help combat fungal infections in the indoor rearing of fireflies, and also will be contributed to a deeper understanding of the diversity of firefly pathogenic fungi.

In order to rich profiles of pathogen fungi that infect fireflies, and further develop the corresponding treatment methods, in this study, *Trichoderma* pathogenic fungi associated with *Pygoluciola* sp. larvae, an extensively cultivated semi-aquatic firefly, were isolated and identified. Next, an effective and safe antifungal approach against the infection was investigated. The findings in this study will add information to the disease profile of fireflies during indoor rearing. The developed methods can be useful in avoiding infection during the breeding of fireflies and even other insects.

## 2. Materials and Methods

### 2.1. Insect Feeding

Larval *Pygoluciola* sp. were kept at the Culture Centre of Fireflies, Ganzhou, Jiangxi Province, China, a large-scale firefly breeding base, according to the center’s protocols for care and feeding with minor modification [6]. In brief, experiments were conducted at 25 °C, 75% humidity, and a 14 h:10 h L:D photoperiod. The fireflies were fed freshwater snail (*Cipangopaludina chinensis*) muscle tissue purchased from local supermarkets every 24 h, and any food residues were removed.

### 2.2. Fungal Isolation, Identification, and Pathogenicity Test

Mummified host firefly samples infected with a fungus were collected during larval rearing. All samples were gently rinsed with sterile water on a clean bench (AirTech, Dalian, China). Sterile scalpels were then used to dissect out tissues containing mycelium of the pathogenic fungi. These were inoculated onto potato dextrose agar (PDA) medium and incubated at 28 °C for five days. Then, if mycelium growth was detected, further isolation and purification were performed using the three-point inoculation method [11]. The purified pathogenic fungi were inoculated on PDA medium for seven days, whereafter mycelia with conidia were picked from the edge of the colonies, stained with lactophenol cotton blue, and viewed under a light microscope (400× magnification) (Primo Star, Zeiss, Jena, Germany). *Trichoderma koningiopsis* was identified according to morphology described in [12,13], and further determined by 18S rDNA as a molecular marker (Supplementary methods in Supplementary Materials). Finally, a spore suspension was prepared for the pathogenicity assay (Supplementary methods in Supplementary Materials).

### 2.3. Screening of Antifungal Agents and Determination of Minimum Inhibitory Concentration

Ciclopirox olamine, nystatin, griseofulvin, terbinafine, and itraconazole are five clinically popular antifungal agents for treating fungal diseases in humans and animals (Supplementary Table S1). The in vitro screening of the five antifungal agents against *T. koningiopsis* ZL01-induced mycosis in *Pygoluciola* sp. was conducted using the dual culture assay [14,15], with minor modifications (Supplementary methods in Supplementary Materials). Subsequently, the minimum inhibitory concentration (MIC) values of the candidate agents were determined, as previously described [16,17]. Briefly, the MIC assay was performed using sterile 96-well plates. Spore suspension (100 μL, approximately 10^7^ CFU/mL) of *T. koningiopsis* ZL01 was precultured in PDB broth for further experiments. Subsequently, 100 μL (3200 μg/mL) of diluted candidate agents was mixed with 100 μL of the broth containing *T. koningiopsis* ZL01 spore. Finally, 2-fold serial dilutions of the antifungal agents were obtained in sterile the broth, including 1600, 800, 400, 200, 100, 50, 25, 12.5, and 6.25 μg/mL, and incubated at 28 °C for 24 h. The MIC was defined as the lowest concentration of an antifungal agent completely inhibiting the growth of the target fungal strain, i.e., no mycelial growth is observable with the naked eye after treatment. Each sample was independently assessed in triplicate.

### 2.4. Biosafety Assessment of Antifungal Agents

Acute oral toxicity and acute contact toxicity tests were used to evaluate the biosafety of the above-mentioned antifungal agents on larval *Pygoluciola* sp. A total of 300 healthy 4th-instar larvae were randomly placed in 30 clean, sterile Petri dishes with moist filter paper (10 individuals per Petri dish). These were divided into five experimental groups: a control group, a nystatin acute oral toxicity group, a DMSO (nystatin solution) acute oral toxicity group, a nystatin acute contact toxicity group, and a DMSO acute contact toxicity group. Each group included six experimental Petri dishes (60 individuals in total per group), covering three biological replicates (20 individuals per two dishes per replicate). Experimentally treated firefly larvae were kept as in Section 2.1, and monitored daily for feeding and mortality. The specific procedures for the acute oral and contact toxicity assays were presented for details in Supplementary methods in Supplementary Materials. The results of each group were analyzed by consolidating the experimental data from all six Petri dishes in that group.

### 2.5. Effect of Nystatin on Firefly Survival Under the Introduction of T. koningiopsis ZL01

In total, 1080 healthy 4th-instar firefly larvae were randomly placed in 18 artificial feeding rearing cages, 60 cm × 18 cm × 40 cm, with 60 individuals per cage (Supplementary Figure S1). Animals were divided into six experimental groups: the control group, *T. koningiopsis* ZL01 treatment group, *T. koningiopsis* ZL01 + drip treatment group at 1 × MIC concentration of nystatin (nystatin drip group), *T. koningiopsis* ZL01 + DMSO control group at the corresponding concentration (DMSO drip group), *T. koningiopsis* ZL01 + spray treatment group at 1 × MIC concentration of nystatin (nystatin spray group) and the *T. koningiopsis* ZL01 + DMSO control group at the corresponding concentration (DMSO spray group). Each group included three artificial feeding cages as three replicates. Body surface washing using sterile water was performed on all individuals before initiation of the experiment. Except for the controls, the individuals in the other five experimental groups were subsequently used to construct *T. koningiopsis* ZL01 mycosis-infected *Pygoluciola* sp. models according to the pathogenicity test method in Section 2.2.

After 12 h, a body surface drip treatment of nystatin for the *T. koningiopsis* ZL01-treated *Pygoluciola* sp. larvae was conducted in the nystatin drip group according to Section 2.4 (similarly, 1× MIC at concentration, 10 μL per individual). Spray treatment of nystatin was also performed in the nystatin spray group (1× MIC at concentration, 10 μL per larva, sprayed individually) with the DMSO drip and spray groups set in parallel. The experimental solution was sprayed from the immediate airspace, approximately 1 cm above the fireflies, using a micropipette with miniature water spray nozzle. Thereafter, all larvae were transferred to their respective rearing cages, treatment continously conducted on the third and sixth day. All larvae were fed normally for nine days according to Section 2.1. Mortality was assessed as described in Section 2.4, and dead individuals were removed immediately.

### 2.6. Statistics

Results are expressed as the mean of three biological replicates ± standard error (S.E.). Multiple comparisons were performed using a One-Way Analysis of Variance (ANOVA) followed by Duncan’s test in Prism 8.0.2 (GraphPad Software Inc., La Jolla, CA, USA). A *p*-value of less than 0.05 was set as the threshold for statistical significance.

## 3. Results

### 3.1. Identification of Fungal Pathogens Infecting Pygoluciola *sp.* Larvae

**Figure 1:**
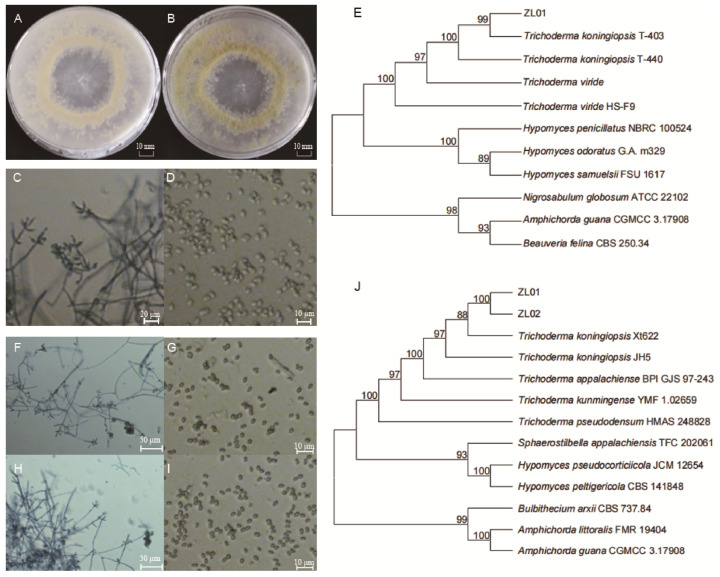
Identification of a fungal pathogen infecting *Pygoluciola* sp. larvae. Colonial morphology of *Trichoderma koningiopsis* ZL01 after incubation for seven days on PDA medium in reverse (**A**) and obverse view (**B**). Mycelial (**C**) and conidial (**D**) morphology of *T. koningiopsis* ZL01. Phylogenetic tree (**E**) constructed from 18S sequences of Hypocrealesc fungi. Mycelial (**F**,**H**) and conidial (**G**,**I**) morphology of *T. koningiopsis* ZL01 and ZL02, respectively. Phylogenetic tree (**J**) constructed from ITS sequences of Hypocrealesc fungi.

A purified strain of the cultured fungus presented colonies with a diameter of approximately 75 mm after seven days of incubation, with a uniform surface texture without any raised areas and an edge with regular, feathery extensions. The mycelium was dense, white, and flocculent (cotton-like), with distinct concentric rings visible in the colony (Figure 1A,B). The strain showed a phialide layer structure, and conidiophores originated from conidial tufts, with short, narrow primary branches that ramified further. Phialides were typically arranged in verticillate clusters of 2–5, rarely solitary, ampulliform in shape with a constricted base, with a length of 4.7–10.1 μm and a width of 2.4–4.5 μm (Figure 1C). The mycelium diameter ranged from 2.5 to 4.0 μm, and the primary conidiophores were approximately 3 μm in width. The conidia were grayish-green, elliptical (ovoid), and smooth-surfaced, with dimensions of 2.5–4 × 2–3 μm (Figure 1D). The amplified 18S rDNA of ZL01 had a sequence length of 1360 bp (Genbank accession No. PX454474) and a 99.41% similarity with *T. koningiopsis* strain T-403 (GenBank accession No. MT544907.1). The BLAST+ 2.17.0 alignment showed the sample cluster with *T. koningiopsis* separate from other *Trichoderma* species (Figure 1E). Thus, the ZL01 strain was identified as *T. koningiopsis* based on morphological and molecular evidence.

Additionally, the pathogenicity test results of *T. koningiopsis* ZL01 showed that the spore suspension successfully infected *Pygoluciola* sp. larvae (Supplementary Figure S2). Specifically, locomotion of the experimental individuals began to slow down after day three, and was accompanied by frequent episodes of death feigning and eventually death. Mycelium could be observed with the naked eye on the body surface after day 10~12, and the mycelium had colonized the larva’s appendages and began quickly spreading to other parts of the body after day 14, while the entire body was fully covered by day 20. Moreover, the re-purified fungal samples presented similar microscopic morphological characteristics after culture (mycelium and conidiospore of ZL01 showed by Figure 1F,G, ZL02 by Figure 1H,I), further determined by ITS sequencing (Figure 1J). Results showed a sequence length of 575 bp (accession No. PX454472 of ZL01 and PX454473 of ZL02), with a 98.09% and 98.35% similarity with *T. koningiopsis* strain Xt622, respectively. Collectively, these results demonstrated that this strain is a pathogenic fungus, *Pygoluciola* sp.

### 3.2. Determination of Antifungul Agents and the Biosafety

**Figure 2:**
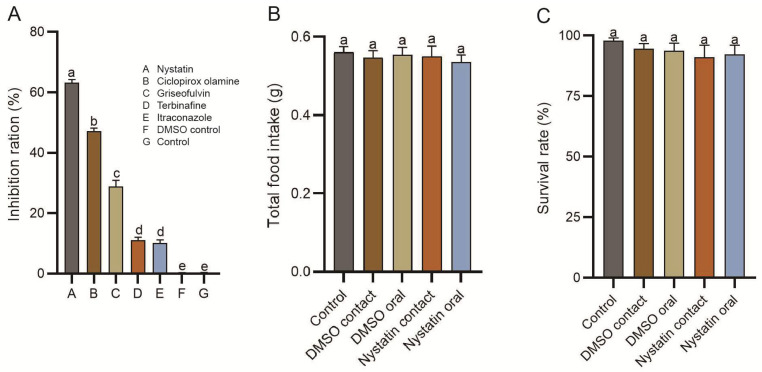
Determination of antifungul agents and the biosafety. In vitro inhibition rate of experimental antifungal candidates on the mycelial growth of *T. koningiopsis* ZL01 in firefly larvae (**A**). Toxicity test of nystatin on *Pygoluciola* sp. larvae by acute contact and oral administration, reflected by the total food intake (**B**) and survival rate (**C**) at the end of experiments (six days). Error bars represent S.E. of biological replications. Different letters above the data columns indicate significant differences at the 0.05 level.

Compared to the control groups, a significant inhibition (*p* < 0.05) of mycelial growth of *T. koningiopsis* ZL01 was observed for all five antifungal agents (Figure 2A). Among these candidates, nystatin exhibited the best antifungal activity against *T. koningiopsis* ZL01 (mycelial growth was inhibited by 63.25 ± 0.36%), followed by ciclopirox olamine (47.19 ± 0.59%), griseofulvin (28.89 ± 0.67%), terbinafine (11.01 ± 0.18%), and itraconazole (10.18 ± 0.39%). Additionally, the MIC values of nystatin, ciclopirox olamine, griseofulvin, terbinafine, and itraconazole against *T. koningiopsis* ZL01 were 25.00 μg/mL, 50.00 μg/mL, 100.00 μg/mL, 400.00 μg/mL, and 400.00 μg/mL, respectively.

Furthermore, nystatin and the corresponding control DMSO showed no significant harmful effects on the *Pygoluciola* sp. larvae in the acute oral and acute contact toxicity tests. In particular, total food intake showed no significant difference among all five groups upon completion of the 6-day experiment, with values of 0.56 ± 0.02 g, 0.54 ± 0.02 g, 0.55 ± 0.02 g, 0.55 ± 0.03 g, and 0.53 ± 0.02 g in the control, DMSO contact, DMSO oral, nystatin contact, and nystatin oral groups, respectively (Figure 2B). Meanwhile, no significant difference was also detected in larval mortality among the five groups, with values of 98.33 ± 1.67%, 95.00 ± 3.33%, 93.33 ± 5.00%, 91.66 ± 6.67%, and 93.33 ± 5.00%, respectively (Figure 2C).

### 3.3. Effects of Nystatin on Survival of a T. koningiopsis ZL01 Infected Larval Population

**Figure 3:**
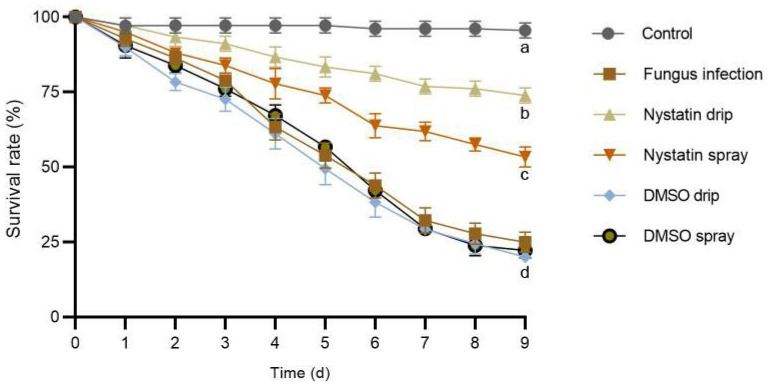
Survival rate dynamics of *Pygoluciola* sp. larvae infected with *T. koningiopsis* ZL01 after nystatin treatment over nine days with different application methods in artificial breeding. Error bars represent the S.E. of biological replicates. Different letters below the data signs indicate significant differences at the 0.05 level. The “d” contains three groups with no significant differences.

An obvious decrease in larval survival was detected in all the experimental groups as time progressed (Figure 3). No significant decrease in survival rate was observed in the control groups (a decrease of 4.45 ± 1.67% from day 0 to day 9). Instead, the *T. koningiopsis* ZL01, *T. koningiopsis* ZL01 + DMSO drip, and *T. koningiopsis* ZL01 + DMSO spray treatment groups showed the largest decrease in survival rate (decreased by 75.00 ± 3.33%, 80.00 ± 1.67%, and 77.78 ± 3.33%, respectively, from day 0–9). The larval survival rate decreased by 26.11 ± 1.67% and 46.67 ± 3.33% in the *T. koningiopsis* ZL01 + nystatin drip and *T. koningiopsis* ZL01 + nystatin spray groups, respectively, from day 0–9. Particularly, the *T. koningiopsis* ZL01 + nystatin drip population consistently retained the highest survival rate of all the experimental groups throughout the experiment. Finally, the survival rate of the *T. koningiopsis* ZL01-treated *Pygoluciola* sp. population reached 25.00 ± 3.33%, 20.00 ± 1.67%, 22.22 ± 3.33%, 73.89 ± 1.67%, and 53.33 ± 3.33% in *T. koningiopsis* ZL01, *T. koningiopsis* ZL01 + DMSO drip, *T. koningiopsis* ZL01 + DMSO spray, *T. koningiopsis* ZL01 + nystatin drip, and *T. koningiopsis* ZL01 + nystatin spray groups, respectively, after nine days.

## 4. Discussion

Although fungal disease outbreaks have not been reported in semi-aquatic fireflies, mycosis has been frequently detected in practical artificial rearing. This may be because water maintenance within the rearing environment triggers fungal spore germination and enhances mycelium development [9]. Thus, water and soil used in artificial breeding of firefly larvae must be pre-sterilized (such as pressured steam at 121 °C for 1 h at least or filter membrane in 0.22-μm pore size) to inactivate fungal spores, decreasing the risk of pathogen infection and transmission. In this study, we documented one species of pathogenic microfungi able to infect the firefly *Pygoluciola* sp. larvae and cause mortality in approximately three days after infection. Previous studies reported that *Trichoderma harzianum* infects the terrestrial firefly, *Pteroptyx bearni,* during the larval stage, causing absolute mortality once the infection was detected [9]. This suggests that *Trichoderma* fungi cause mycosis in firefly larvae, although more extensive studies are needed from additional firefly lineages. Due to the intimate association between microbes and their hosts, many studies have focused on gut microbes in fireflies in recent years [18,19]. Unfortunately, pathogenic fungi have not received sufficient attention, presenting a major hidden risk in the artificial rearing of fireflies.

Several chemical compounds with antifungal activity, such as tegosept, benzoic acid, formalin, potassium sorbate, and sodium propionate, can decrease fungal growth in rearing systems for insects when added as additives in artificial diets [19,20]. However, it has been observed that these chemical compounds repel larval *Pygoluciola* sp. when added to their diets. Therefore, in this study, five common antifungal chemicals widely used in the treatment of fungal infections of skin and mucous of human were tested as candidates suitable for drip and spray treatments of firefly larvae. The results showed that nystatin has the highest antifungal efficacy and the lowest MIC value against *T. koningiopsis* ZL01 growth in vitro. In addition, effective antifungal agents for insect rearing must not only suppress mold growth but also be safe [21]. Here, food intake and survival rate of the larvae are considered two key indicators in drug safety evaluation [21]. Toxicity tests demonstrated that these two parameters were not significantly affected by acute oral and contact treatment with nystatin, while the corresponding solvent (DMSO) exhibited no adverse effect in *Drosophila melanogaster* [22] in previous studies, suggesting the safety of nystatin and DMSO use in firefly larvae. Notably, this remains to be substantiated by experimental indicators on broader temporal scales, such as egg production, time to adult emergence, and initiation of egg laying [21,23].

In the present study, *T. koningiopsis* ZL01 was introduced into a larval *Pygoluciola* sp. population to construct mycosis models used to evaluate the therapeutic efficacy of nystatin in artificial rearing conditions. During treatment over nine days, nystatin obviously reduced the mortality of the larvae infected with *T. koningiopsis* ZL01 (approximately three-fold the survival rate of untreated groups at the end of the experiment), a trend that was maintained compared with the control groups. The drip treatment of nystatin showed the highest survival rate, more than 20% compared to treatment with nystatin spray by the end of the experiment. Collectively, these results advocate for the therapeutic efficacy of nystatin drip treatment on firefly mycosis under artificial rearing conditions. As a classic clinical antifungal medication, the industrial production of nystatin has been well-established for decades [24,25], ensuring a sufficient market supply at a surprisingly low cost (one gram of nystatin powder ($4) can cover millions of firefly individuals per treatment (1 × MIC/25.00 μg/mL at concentration and dripping 10 μL per individual)). The ecological, cultural, and economic significance of fireflies [26,27] promoted the demand for artificial feeding, highlighting the application value of this study.

## 5. Conclusions

This study reported on the fungi that are associated with the larval *Pygoluciola* sp., providing information to the disease profile of semi-aquatic fireflies in artificial rearing conditions. We further developed a nystatin-based antifungal approach against *T. koningiopsis* ZL01 infection in *Pygoluciola* sp. larvae during indoor rearing. This will be useful for the decreasing infection rate of fungi towards the fireflies’ larvae in the laboratory setting.

## Data Availability

The original contributions presented in this study are included in the article/Supplementary Materials. Further inquiries can be directed to the corresponding authors.

## Supplementary Materials

The following supporting information can be downloaded at https://www.mdpi.com/article/10.3390/insects16121193/s1. Figure S1. Schematic diagram of indoor breeding device for *Pygoluciola* sp. A: Diagrammatic sketch (five view) of outside and internal habitat arrangement of the artificial feeding cages of semi-aquatic firefly *Pygoluciola* sp. larvae. The rigid plastic rearing cages (60 cm × 18 cm × 40 cm) with a opening top was used as the rearing apparatus for larval *Pygoluciola* sp., with smooth walls. The cages were disinfected prior to use, then placed a 3–5 cm thick layer of sterilized clay at the bottom, followed by a layer of moss and sealing with a 40-mesh insect-proof net around magnetic strips. B: Layout drawings (three view) of the artificial feeding system, including rearing racks, spraying system, and drainage system, except for the artificial feeding cages. Rearing rack: A hollow-frame iron rack (240 cm × 25 cm × 150 cm) included three tiers with an interlayer spacing of 45 cm. Spraying system: A pressurized water pump was used to draw water from a storage tank into the spraying system installed above the cages. The water in the form of aerated mist was then dispersed through high-pressure atomizing nozzles, maintaining saturated soil moisture. Drainage system: An opening was created on the front side of the rearing cages to allow wastewater discharge, thereby preventing the accumulation of visibly distinct standing water. The temperature kept at 25 °C, 75% humidity, and an L:D = 14 h:10 h photoperiod provided by LED light source installed on above the rearing cage. Figure S2. Dynamics changes in surface of *Pygoluciola* sp. larvae infected by *T. koningiopsis* ZL01. A: 3 d, B: 10 d, C: 12 d, D: 14 d, E: 16 d, and F: 20 d after inoculation. Table S1. Detailed information of five candidate antimicrobial agents used in this study. References [6,14,15,28] are cited in the supplementary materials.
