# Supplementary material for: Pathogen Identification and Treatment of Trichoderma koningiopsis ZL01 Mycosis in Firefly Pygoluciola sp. (Coleoptera: Lampyridae)
Source: Insects. 2025 Nov 23;16(12):1193. doi: 10.3390/insects16121193 (PMC12734182; doi:10.3390/insects16121193)
Supplement: Supplementary file 1 [file insects-16-01193-s001.zip › insects-3981833-supplementary.pdf]

Supplementary Materials

Supplementary methods

1. Determination of pathogenic fungi using molecular biology

The fungal mycelium was transfered into 2-mL Eppendorf tube with glass beads in size of 2-mm diameter, vibrated by 10 min. Genomic DNA of pathogenic fungi was extracted using UNlQ-10 Column Fungal Genomic DNA Isolation Kit (Sangon Biotech，China). The 18S ribosomal DNA (rDNA) of the fungi was amplified with universal primers NS1 (GTAGTCATATGCTTGTCTC) and NS6 (GCATCACAGACCTGTTATTGCCTC). PCR reaction system: 2 × Mix 12.5 μL, DNA template 2 μL, NS1 and NS6 primers 1.0 μL for each. PCR reaction conditions: 94 ℃ for 4 min, 95 ℃ for 55 s, 58 ℃ for 50 s, 72 ℃ for 90 s, final extension at 72 ℃ for 10 min after 35 cycles. PCR products were detected by 2.0% agarose electrophoresis, and then Sanger sequencing was performed by Sangon Biotech Co., Ltd (Shanghai, China). The sequencing results were searched in the NCBI database using BLAST for homology alignment. Fungal species with more than 98% concordance and the best blast were used as target species. Phylogenetic tree was reconstructed using MEGA6 software [1].

2. Preparation of pathogenic fungal spore suspension and pathogenicity test

The purified mycelia were inoculated on PDA medium and cultured in a constant temperature incubator (Shanghai Hengyi, China) at 28 °C. When mature spores formed, the mycelia and conidia were transferred using an inoculation shovel into a 50-mL tube containing sterile water with 0.5% Tween-80 and glass beads. The tube was placed on a shaker (Scientific Industries, America), oscillated for 1–2 minutes until the conidia were completely dispersed and homogenized. The spore suspension was then filtered through two layers of sterile gauze. A 10-μL aliquot was aspirated with a pipette, and the spore concentration was determined using a hemocytometer. The concentration was adjusted to a final value of 1.0×10^7 CFU/mL, sealed and stored at 4 °C for subsequent experiments.

Next, 50 individuals of healthy 4th instar *Pygoluciola* sp. larvae were selected, rinsed with sterile water, and then immersed in the prepared spore suspension for 30 s. After air-drying, the larvae were transferred to petri dishes with sterile moist filter paper, reared under the same conditions as Section 2.1 and monitored every 48 hours under a stereomicroscope. Upon the visual appearance of extensive mycelial coverage, the larvae were collected. Fungal mycelial were then inoculated on PDA medium to detect colony morphology and structure, followed by determination of morphology and molecular biology. An additional molecular markers ITS was introduced to further determine the taxonomic classification of the strain by cross-validation with the results of 18S rDNA. Universal primers of ITS used here are ITS1 (TCCGTAGGTGAACCTGCGG) and ITS4 (TCCTCCGCTTATTGATATGC).

3. Screening of antifungal agents

The powder of five candidate antimicrobial agents (i.e., ciclopirox olamine, nystatin, griseofulvin, terbinafine, and itraconazole) was dissolved in dimethyl sulfoxide (DMSO) to prepare stock solutions. After filter sterilization using 0.22-μm sterile microporous membranes, the stock solutions were adjusted to a working concentration (6.4 mg/mL, as similarly used in [2]). An confrontation assay was then conducted with the pathogenic fungus *T. koningiopsis* ZL01: a clean, sterile Oxford cup and the mycelia of strain ZL01 were placed on opposite sides on a PDA plate, with the distance between the center of the Oxford cup and the inoculation point uniformly set at 4.1 cm. A volume of 200 μL of each antifungal agent was added to the corresponding Oxford cup. A blank control (with an equal volume of sterile water) and a negative control (with an equal volume of DMSO solution) were included. The experiments were independently repeated three times as three biological replicates. All plates were incubated in a constant temperature incubato at 28 °C in darkness for 5 days. After that, semidiameter of the colony was measured along the straight line connecting the center of the Oxford cup and the inoculation point using a vernier caliper. The growth inhibition rate of *T. koningiopsis* ZL01 was calculated according to the following formula [3]: Growth inhibition rate (%) = (Control colony radius −

Treated colony radius)/Control colony radius × 100%.

4. Acute oral toxicity and acute contact toxicity test of *Pygoluciola* sp. larvae

The acute oral toxicity test: Prior to the experiment, firefly individuals were subjected to a 48-hour starvation period to avoid interference caused by the existing foods. After that, *C. chinensis* muscle (0.50 g per petri dish, common foods for *Pygoluciola* sp. larvae) was immersed in 1× MIC of nystatin for 2 minutes (set the corresponding concentration of DMSO solution as negative control and blank control of sterile water). The treated *C. chinensis* muscle was then used to feed *Pygoluciola* sp. larvae, thereby establishing control, the acute nystatin oral toxicity, and the acute DMSO oral toxicity groups, respectively.

The acute contact toxicity test: The pretreatment procedures were identical to that in the acute oral toxicity test. Next, a 10 μL droplet of nystatin solution at 1×MIC concentration and DMSO solution was administered topically to the dorsal plate of each firefly individual using a micropipette. After the larval surfaces were dry, they were transferred to sterile petri dishes with moist filter paper, and fed using untreated *C. chinensis* muscle. Finally, the acute nystatin contact toxicity and the acute DMSO contact toxicity groups were established, respectively.

The above all the five experimental groups were kept for five days according to rearing methods in Section 2.1. The food and filter paper were replaced every 24 hours, and the residues were collected and weighed. The weight of daily consumed food was calculated according to our previous studies: initial weight + weight of the absorbed water for 24 h – weight of the remaining foods [4]. The data was analyzed at the end of the experiments by incorporating all observational data collected over the 6-day period.

To obtain survival rate for each experimental group at the end of the test, dead individuals was continously collected during the experimental period. Death of individuals was defined by the following criteria: rigid body with no movement, darkened abdominal coloration, and no response to external stimuli resulted by insect pin. To avoid a death-feigning behavior, larvae exhibiting a curled posture were

examined under a stereomicroscope for any movement of appendages, and gently touched with sterile forceps. The confirmed dead larvae were then transferred to 2-mL sterile centrifuge tubes and stored at -80 ℃.

Reference

1. Tamura, K.; Stecher, G.; Peterson, D.; Filipski, A.; Kumar, S. MEGA6: Molecular evo lutionary genetics analysis version 6.0. *Mol Biol Evol* **2013**, *30*, 2725-2729, https://doi.org/10.1093/molbev/mst197.
2. Tang, C.; Kong, X.; Jansen, J.; Vossgroene, K.; Vu, T.L.A.; Oberheitmann, B.; Tehupe iory‐Kooreman, M.; Zhou, S.; Zhou, X.; Tsui, C.K.; et al. Utility of MALDI‐ToF MS for recognition and antifungal susceptibility of Nannizzia, an underestimated group of Dermatophytes. *Mycoses* **2025**, *68*, https://doi.org/10.1111/myc.70117.
3. Holkar, S.K.; Ghotgalkar, P.S.; Lodha, T.D.; Bhanbhane, V.C.; Shewale, S.A.; Markad, H.; Shabeer, A.T.P.; Saha, S. Biocontrol potential of endophytic fungi originated from grapevine leaves for management of anthracnose disease caused by *Colletotrichum glo eosporioides*. *3 Biotech* **2023**, *13*, 258, https://doi.org/10.1007/s13205-023-03675-z.
4. Yang, L.Y.; Tang, D.R.; Li, F.X.; Luo, S.Q.; Cao, C.Q.; Zhang, Q.L. Larval feeding h abits of five firefly species across aquatic, semi-aquatic, and terrestrial lineages. *Insects* **2024**, *15*, https://doi.org/10.3390/insects15121004.

Supplementary tables and figures

Table S1. Detailed information of five candidate antimicrobial agents used in this study.

Candidates	Mechanism of action	Source
Ciclopirox olamine	Disrupt fungal cell membrane integrity	Hubei WeiShi Reagent Co., Ltd., Wuhan, China, product code: CP001798
Nystatin	Bind to ergosterol in the membrane, interfering with fungal metabolism and increasing membrane permeability	YuanYe Bio-Technology Co., Ltd., Shanghai, China, product code: S17029
Riseofulvin	Interfere with fungal microtubule structure	YuanYe Bio-Technology Co., Ltd., Shanghai, China, product code: B25392
Terbinafine	Inhibit fungal squalene epoxidase activity	YuanYe Bio-Technology Co., Ltd., Shanghai, China, product code: B34035
Itraconazole	Inhibit ergosterol biosynthesis in the membrane	YuanYe Bio-Technology Co., Ltd., Shanghai, China, product code: B25558

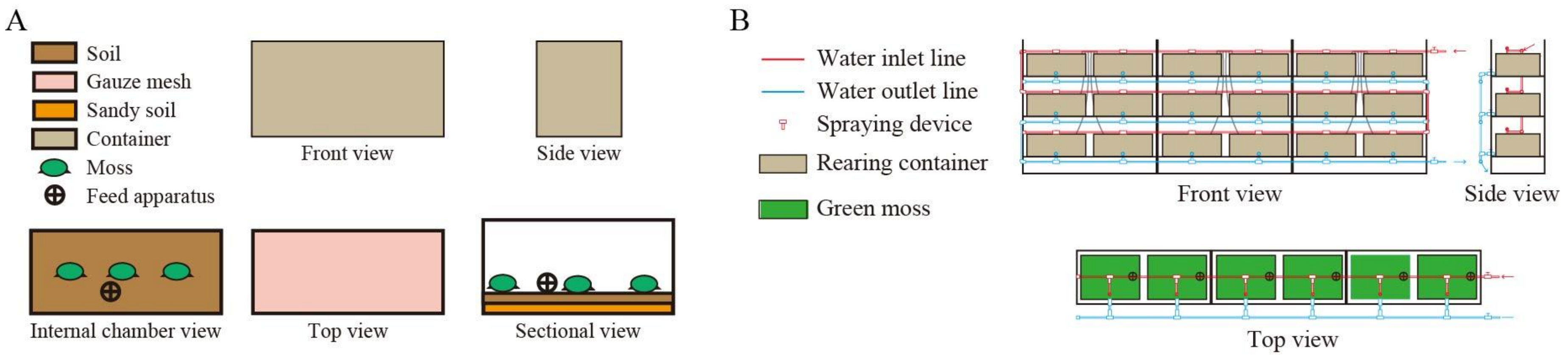

Figure S1. Schematic diagram of indoor breeding device for *Pygoluciola* sp.. A: Diagrammatic sketch (five view) of outside and internal habitat arrangement of the artificial feeding cages of semi-aquatic firefly *Pygoluciola* sp larvae. The rigid plastic rearing cages (60 cm × 18 cm × 40 cm) with a opening top was used as the rearing apparatus for larval *Pygoluciola* sp., with smooth walls. The cages were disinfected prior to use, then placed a 3–5 cm thick layer of sterilized clay at the bottom, followed by a layer of moss and sealing with a 40-mesh insect-proof net around magnetic strips. B: Layout drawings (three view) of the artificial feeding system, including rearing racks, spraying system, and drainage system, except for the artificial feeding cages. Rearing rack: A hollow-frame iron rack (240 cm × 25 cm × 150 cm) included three tiers with an interlayer spacing of 45 cm. Spraying system: A pressurized water pump was used to draw water from a storage tank into the spraying system installed above the cages. The water in the form of aerated mist was then dispersed through high-pressure atomizing nozzles, maintaining saturated soil moisture. Drainage system: An opening was created on the front side of the rearing cages to allow wastewater discharge, thereby preventing the accumulation of visibly distinct standing water. The temperature kept at 25 ℃, 75% humidity, and an L: D=14 h: 10 h photoperiod provided by LED light source installed on above the rearing cage.

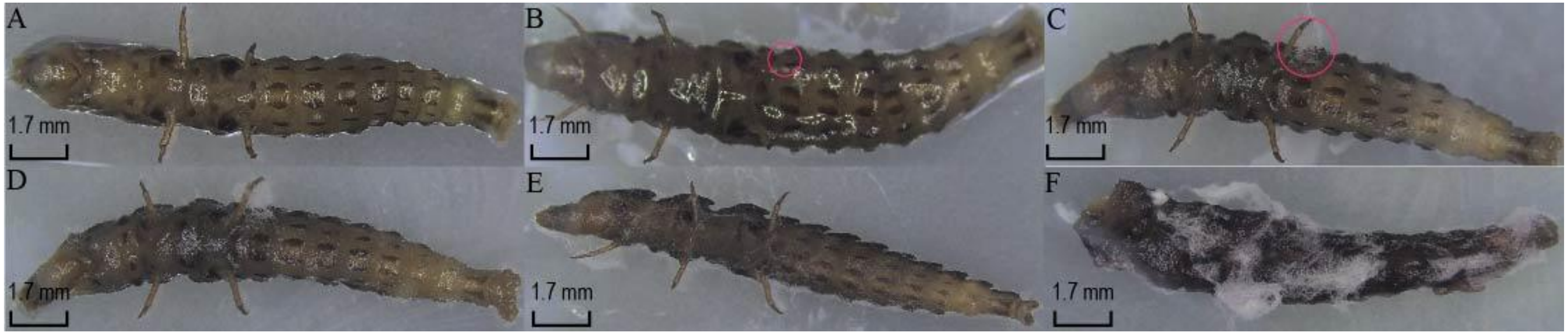

Figure S2. Dynamics changes in surface of *Pygoluciola* sp. larvae infected by *T. koningiopsis* ZL01. A: 3 d, B: 10 d, C: 12 d, D: 14 d, E: 16 d, and F: 20 d after inoculation.
